# The impact of regular physical activity on vertebral fractures: Korean nationwide population-based cohort study

**DOI:** 10.1186/s12891-024-08179-2

**Published:** 2025-01-22

**Authors:** Sangsoo Han, Sungwoo Choi, Hae-Dong Jang, Jiwon Park, Kyungdo Han, Sangun Nah, Dong Hun Suh, Dong Sub Im, Jae-Young Hong

**Affiliations:** 1 Department of Emergency Medicine, Soonchunhyang University Bucheon Hospital, 170 Jomaru-ro, Bucheon, 14584 Republic of Korea; 2 Department of Orthopaedic Surgery, Soonchunhyang University Bucheon Hospital, 170 Jomaru-ro, Bucheon, 14584 Republic of Korea; 3 Department of Orthopedics, Korea University Hospital, 123, Jeokgeum-ro, Danwon-gu, Ansan, Ansan-si, 15355 Gyeonggi-do Republic of Korea; 4 Department of Statistics and Actuarial Science, Soongsil University, 369 Sangdo-ro, Dongjak- gu, Seoul, 06978 Republic of Korea; 5 Department of Orthopedic Surgery, Gil Medical Center College of Medicine, University of Gachon, Incheon, Republic of Korea

**Keywords:** Physical activity, Fracture, Vertebrae, Health surveys, Exercise

## Abstract

**Background:**

Vertebral fractures are a common medical issue. Physical activity helps improve bone health and manage the risk of fractures. However, much controversy exists about the association between physical activity and vertebral fractures. Therefore, we aimed to investigate the association between changes in regular physical activity (RPA) and vertebral fractures.

**Methods:**

This study was a nationwide, observational cohort study based on claim data from the Korean National Health Insurance Service. Physical activity level was obtained from self-reported questionnaires from 2009 to 2012. Participants were divided depending on their levels of physical activity and the occurrence of vertebral fractures from 2013 to 2016 was recorded.

**Results:**

The group with sufficient RPA, compared to the Insufficient group (incidence rate showed a decrease of 1.93/1,000 PY; adjusted HR (aHR): 0.83; 95% CI: 0.81–0.84), had a reduced risk of vertebral fracture. From the perspective of RPA changes, the Continuous Sufficient group had an aHR of 0.74, 95% CI 0.72–0.76, and the Increased group had an aHR of 0.87, 95% CI 0.85–0.88. The Decreased group had an aHR of 0.94, 95% CI 0.92–0.95.

**Conclusion:**

The degree of RPA could reduce the risk of vertebral fracture. Continuous sufficient RPA helped lower the risk of vertebral fracture.

**Supplementary Information:**

The online version contains supplementary material available at 10.1186/s12891-024-08179-2.

## Introduction

Vertebral fracture is a common osteoporotic fracture and a significant medical issue worldwide. Vertebral fractures tend to be more common in older adults, and in women [1, 2]. Worldwide, approximately 1.4 million people suffer vertebral fractures every year, and approximately 40% of women are reported to experience vertebral compression fractures during their lifetime [3–5].

Vertebral fractures are also an independent risk factor for additional fractures and can cause pain, physical deformity, reduced physical activity, socioeconomic isolation, depression, and even mortality [6–8]. Additionally, it is reported that if a vertebral fracture occurs, subsequent vertebral fractures are likely to occur, and the 1-year mortality rate is high, so the first fracture event should be considered an early warning [9, 10]. As the population ages, vertebral fractures continue to increase, which is not only an individual burden but also a socioeconomic burden, such as increased medical cost, morbidity, immobilization and healthcare system, contributing to a global public health problem [9, 11].

Physical activity helps improve bone health and manages the risk of developing fractures [12]. In addition, physical activity helps prevent fractures by improving functional aspects, preventing falls that pose a risk of fractures, and influencing bone remodeling through mechanical stimulation of bones [13]. However, some studies have shown that physical activity does not significantly prevent vertebral fractures [14, 15]. On the other hand, some studies have reported that strengthening back muscles through moderate to vigorous physical activity reduces vertebral fractures [16, 17]. As such, much controversy exists about the association between physical activity and vertebral fractures. Therefore, we aimed to investigate the relationship between regular physical activity (RPA) according to decision matrix by public health guidelines, and vertebral fractures [18]. To the best of our knowledge, there is no published report on this topic.

This study analyzed nationwide population-based data collected in Korea from 2009 to 2012. We wanted to analyze the relationship between the degree of RPA and vertebral fractures and to elucidate the effect of variables such as gender, age, lifestyle (e.g., smoking), and prior fracture.

## Materials and methods

### Participants and data

This was a population-based, nationwide, observational cohort study based on claim data from the Korean National Health Insurance Service (KNHIS) [19]. Korea’s National Health Insurance is mandatory for all citizens, and more than 97% of the country’s citizens receive this benefit. The other 3% of the citizens are involved in the Medical Aid Program. People enrolled in KNHIS pay 30% of total medical costs, and medical service providers request that KNHIS pay the remaining amount. It covers all medical treatments other than those resulting from cosmetic procedures and traffic accidents. Additionally, KNHIS conducts regular checkups every 1–2 years for all Korean citizens over the age of 40 who have insurance and for all citizens over the age of 20 who are employed. During this examination, physical measurements, basic blood tests, lifestyle habits, and symptoms are reported through questionnaires. Such information is collected through all hospitals and medical institutions, and since the KNHIS database contains sensitive information such as regional characteristics, examination results, diagnosis, and prescription records, it is carefully controlled by the institution.

**Fig. 1:**
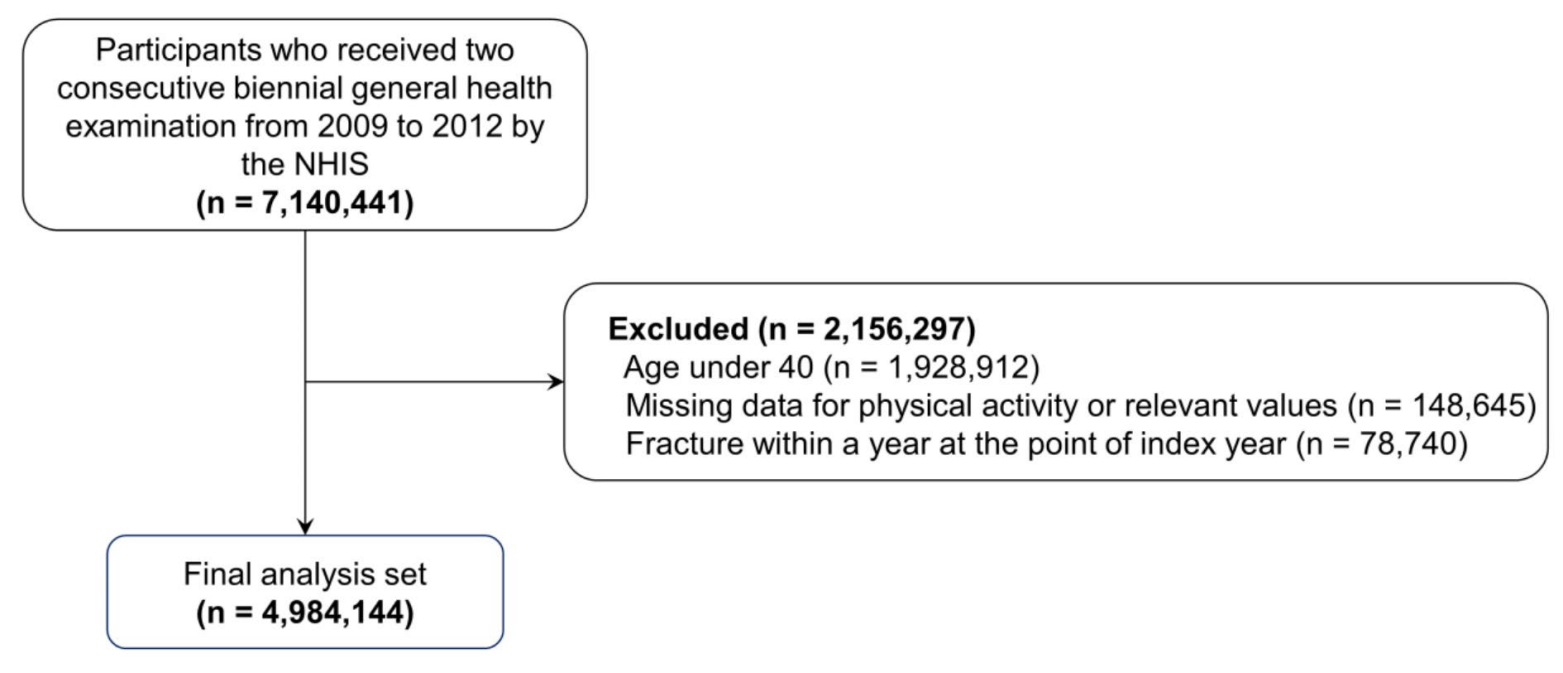
Flowchart of participation in National Health Insurance Service from 2009 to 2012

We enrolled 7,140,441 adults who underwent consecutive biennial KNIHS examinations over two periods: 2009 and 2010, or 2011 and 2012. Among them, 1,928,912 people under 40 years of age were excluded, and 148,645 people with an absence of physical activity or related values were excluded. Additionally, 78,740 people with a history of fracture within one year from the base year were excluded. Finally, 4,984,144 people were enrolled in the final analysis set. (Fig. 1)

The NHIS Institutional Review Board approved the study protocol, and informed consent was waived because NHIS data are anonymous. The study protocol was approved by the Institutional Review Board of Korea University Hospital (Ansan, South Korea; approval no. 2020AS0030).

### Definition of regular physical activity

Participants shared their physical activity levels and other lifestyle habits through self-reported questionnaires. Physical activity was measured using the International Physical Activity Questionnaire (IPAQ) short version [20]. The IPAQ confirmed the frequency and duration of physical activity over the previous seven days through 7 items. Physical activities such as walking, dancing, and gardening for more than 30 min a day were defined as moderate activity, while activities such as running, rapid cycling, and aerobics for more than 20 min a day were defined as vigorous activity, and the amount of physical activity per week was also analyzed. In addition, regular physical activity was defined as vigorous activity more than three times per week or moderate activity more than five times per week [21]. Based on this, we decided to modify the RPA depending on how it changed over time. If the activity continued to be lower than the RPA criteria during the two regular checkups, we categorized those participants as the Continuous Insufficient group (RPA criteria were not satisfied for both checkups). If the activity was less than the RPA criteria at the first checkup but satisfied the criteria at the later checkup, we defined these participants as part of the Increased group (RPA criteria were satisfied only for a later checkup but not for the first checkup). Those who initially satisfied the RPA level, but had decreased at the later checkup to below the standard amount were defined as the Decreased group (RPA criteria were satisfied only for the first checkup but not for the later checkup). When the activity level continuously satisfied the RPA criteria, these patients were delegated to the Continuously Sufficient group (RPA criteria were satisfied for both checkups).

### Vertebral fracture

This study collected and analyzed data on KNHIS’ medical claim records from January 1, 2013, to December 31, 2016. KNHIS’s hospitalization records and the International Classification of Diseases Tenth Revision (ICD-10) codes were checked. A vertebral fracture was defined as an outpatient visit with the following diagnosis codes more than twice within 12 months: S12.0, S12.1, S12.2, S22.0, S22.1, S32.0, M48.4, and M48.5 [22, 23].

### Variables for adjustment and subgroup analysis

Socioeconomic data such as gender, age, and household income were collected through KNHIS medical insurance claim data, and information about comorbidities was also collected. KNHIS’s national health examination data include lifestyle habits such as smoking and drinking, clinical data such as body mass index (BMI), blood pressure, and renal function, and laboratory test results such as cholesterol levels and blood sugar. Additionally, study participants were divided into subgroups according to age (< 65 years or ≥ 65 years) and BMI (< 25% or ≥ 25%). Those in the bottom 20th percentile of household income were defined as low-income, and prior fractures were defined as any fractures that occurred 2–3 years before the index year through self-reporting through a questionnaire. Diabetes was defined as present in those patients who had E11-E14 among the ICD-10 codes and who were prescribed diabetes medication or had a fasting blood sugar > 126 mg/dL. Hypertension was defined as present in those patients who had systolic blood pressure ≥ 140 mmHg, diastolic blood pressure ≥ 90 mm Hg, or I10-I13 codes appearing more than once a year or who were prescribed high blood pressure medication with an I15 cod. Dyslipidemia was defined as total cholesterol level ≥ 240 mg/dL or present in those patients who were prescribed hyperlipidemia medication with an E78 diagnosis code more than once a year, and chronic kidney disease (CKD) was defined as glomerular filtration rate < 60 mL/min/1.76m^2^. It was defined based on ICD codes validated in previous studies [24, 25].

### Statistical analysis

Student’s t-test was performed to analyze continuous variables and the chi-squared test was performed to analyze categorical variables. Hazard ratios (HRs) were calculated along with 95% confidence intervals (CI) for vertebral fracture according to RPA using Cox regression analysis. The incidence rate (IR) is the outcome rate per 1,000 person-years (PY) divided by the total vertebral fracture number. A Cox proportional hazards regression model was used to analyze the relationship between vertebral fractures and changes in RPA. Four models were set up to analyze covariates related to vertebral fractures. Model 1 is unadjusted, Model 2 is adjusted for age and sex, Model 3 is additionally adjusted for smoking, drinking, and household income, and Model 4 is additionally adjusted for BMI, diabetes, and prior fracture.

Hazard ratio (HR) was obtained through Cox regression in various subgroups, to analyze the relationship between vertebral fractures and changes in RPA. We analyzed patients by subgrouping them according to age, gender, BMI, household income, smoking status, alcohol consumption, comorbidities, and previous history of fracture. Statistical analyses were performed using an SAS software program (ver. 9.3; SAS Institute, Cary, NC, USA). P-values < 0.05 were considered statistically significant.

## Results

### General characteristics

The average age of the 4,984,144 participants was 54.9 ± 10.1 years, of whom 2,659,029 (53.3%) were male. Among all participants, 3,303,504 (66.3%) had insufficient continuous physical activity, and 583,561 (11.7%) had decreased physical activity. On the other hand, 650,888 (13.1%) had an increase in activity, and 446,191 (9.0%) had continuous sufficient activity. Mean age, BMI, the proportion of men, participants over 65 years, high BMI over 25, smoking, drinking, low household income, comorbidities, and history of previous fractures showed significant differences in each group (all p-values < 0.001). There were 88,261 (2.67%) new vertebral fractures during the observation period in the Continuous Insufficient group, 14,302 (2.45%) in the Decreased group, 13,193 (2.03%) in the Increased group, and 6,933 (1.55%) in the Continuous Sufficient group (p-value < 0.001) (Table 1).

**Table 1:** General characteristics of participants according to change in regular physical activity

	Physical activity				
	Continuous insufficient (*n* = 3,303,504)	Decreased (*n* = 583,561)	Increased (*n* = 650,888)	Continuous sufficient (*n* = 446,191)	*P*-value
Age, years	54.7 ± 10.53	56.0 ± 10.18	55.0 ± 9.91	55.1 ± 9.67	< 0.001
≥ 65 years	623,067 (18.86)	124,656 (21.36)	120,002 (18.44)	81,934 (18.36)	< 0.001
Male sex, n (%)	1,687,642 (51.09)	322,733 (55.3)	360,244 (55.35)	288,410 (64.64)	< 0.001
BMI, kg/m^2^	23.94 ± 3.05	24.16 ± 2.93	24.04 ± 2.88	24.16 ± 2.76	< 0.001
≥ 25 (%)	1,120,785 (33.93)	212,040 (36.34)	223,493 (34.34)	159,046 (35.65)	< 0.001
Current smoker, n (%)	701,111 (21.22)	105,362 (18.06)	116,059 (17.83)	76,412 (17.13)	< 0.001
Current drinker, n (%)	1,391,493 (42.12)	250,991 (43.01)	293,956 (45.16)	231,673 (51.92)	< 0.001
Low-income, n (%)	679,813 (20.58)	121,334 (20.79)	137,779 (21.17)	82,003 (18.4)	< 0.001
Comorbidities, n (%)					
Hypertension	1,094,622 (33.14)	212,878 (36.48)	224,188 (34.44)	158,971 (35.63)	< 0.001
Diabetes mellitus	378,394 (11.45)	79,911 (13.69)	82,294 (12.64)	59,516 (13.34)	< 0.001
Dyslipidemia	820,600 (24.84)	156,172 (26.76)	166,169 (25.53)	114,208 (25.6)	< 0.001
CKD	203,313 (6.15)	38,170 (6.54)	40,453 (6.22)	28,153 (6.31)	< 0.001
Urban region, n (%)	1,429,941 (43.3)	266,785 (45.7)	305,875 (47.0)	223,557 (50.1)	< 0.001
Previous Fracture, n (%)	99,987 (3.03)	17,532 (2.69)	17,376 (2.98)	10,148 (2.27)	< 0.001
Vertebral Fracture, n (%)	88,261(2.67)	14,302(2.45)	13,193(2.03)	6,933(1.55)	< 0.001

Values are expressed as the mean ± SD or number (proportion)

BMI, Body Mass Index; CKD, chronic kidney disease

*P* < 0.05 was taken to indicate statistical significance

### Regular physical activity and vertebral fracture

During a total follow-up period of over 20 million PY, 122,689 vertebral fracture events occurred. Multivariable adjustment was performed, and in Model 4, data were adjusted for age, sex, smoking status, alcohol consumption, household income, body mass index, diabetes, and prior fracture. The group with sufficient RPA had a significantly reduced risk of vertebral fracture (Table 2) compared to the group with insufficient RPA (incidence rate showed a decrease of 1.93/1,000 PY; adjusted HR (aHR) was 0.83; 95% CI: 0.81–0.84).

**Table 2:** Risk of vertebral fracture according to regular physical activity

Regular PA	Fracture events (*n*)	Total FU duration (PY)	IR (per 1,000 PY)	Hazard ratio (95% CI)			
				Model 1	Model 2	Model 3	Model 4
Insufficient	102,563	16,498,793.4	6.22	1	1	1	1
Sufficient	20,126	4,694,649.2	4.29	0.69	0.81	0.82	0.83
				(0.68–0.7)	(0.79–0.82)	(0.8–0.83)	(0.81–0.84)

FU, follow-up; PY, person-year; IR, incidence rate; CI, confidence interval; PA, physical activity

Incidence rate = Fracture event/total follow-up duration

Model 1: Non-adjusted

Model 2: Adjusted for age and sex

Model 3: Adjusted for age, sex, smoking status, alcohol consumption and household income

Model 4: Adjusted for age, sex, smoking status, alcohol consumption, household income, body mass index, diabetes and prior fracture

### Change in RPA and vertebral fracture

To analyze the association between vertebral fractures and changes in RPA, we performed Cox regression to obtain HRs. A reference group is a Continuous Insufficient group. In model 4, the Continuous Sufficient group had an adjusted HR of 0.74, 95% CI 0.72–0.76, and the Increased group had an aHR of 0.87, 95% CI 0.85–0.88, indicating a significantly low risk of vertebral fracture. Additionally, the Decreased group had an aHR of 0.94, 95% CI 0.92–0.95 (Table 3).

**Table 3:** Hazard ratios for vertebral fracture according to change in regular physical activity

Regular PA	Fracture events (*n*)	Total FU duration (PY)	IR (per 1,000 PY)	Hazard ratio (95% CI)			
				Model 1	Model 2	Model 3	Model 4
Continuous insufficient	88,261	14,014,911.3	6.30	1	1	1	1
Decreased	14,302	2,483,882.1	5.76	0.91	0.92	0.93	0.94
				(0.9–0.93)	(0.91–0.94)	(0.91–0.95)	(0.92–0.95)
Increased	13,193	2,779,982.8	4.75	0.75	0.85	0.86	0.87
				(0.74–0.77)	(0.83–0.87)	(0.84–0.87)	(0.85–0.88)
Continuous sufficient	6,933	1,914,666.5	3.62	0.58	0.71	0.73	0.74
				(0.56–0.59)	(0.7–0.73)	(0.71–0.74)	(0.72–0.76)

FU, follow-up; PY, person-year; IR, incidence rate; CI, confidence interval; PA, physical activity

Incidence rate = Fracture event/total follow-up duration

Model 1: Non-adjusted

Model 2: Adjusted for age and sex

Model 3: Adjusted for age, sex, smoking status, alcohol consumption and household income

Model 4: Adjusted for age, sex, smoking status, alcohol consumption, household income, body mass index, diabetes and prior fracture

### Subgroup analysis according to change in RPA

**Fig. 2:**
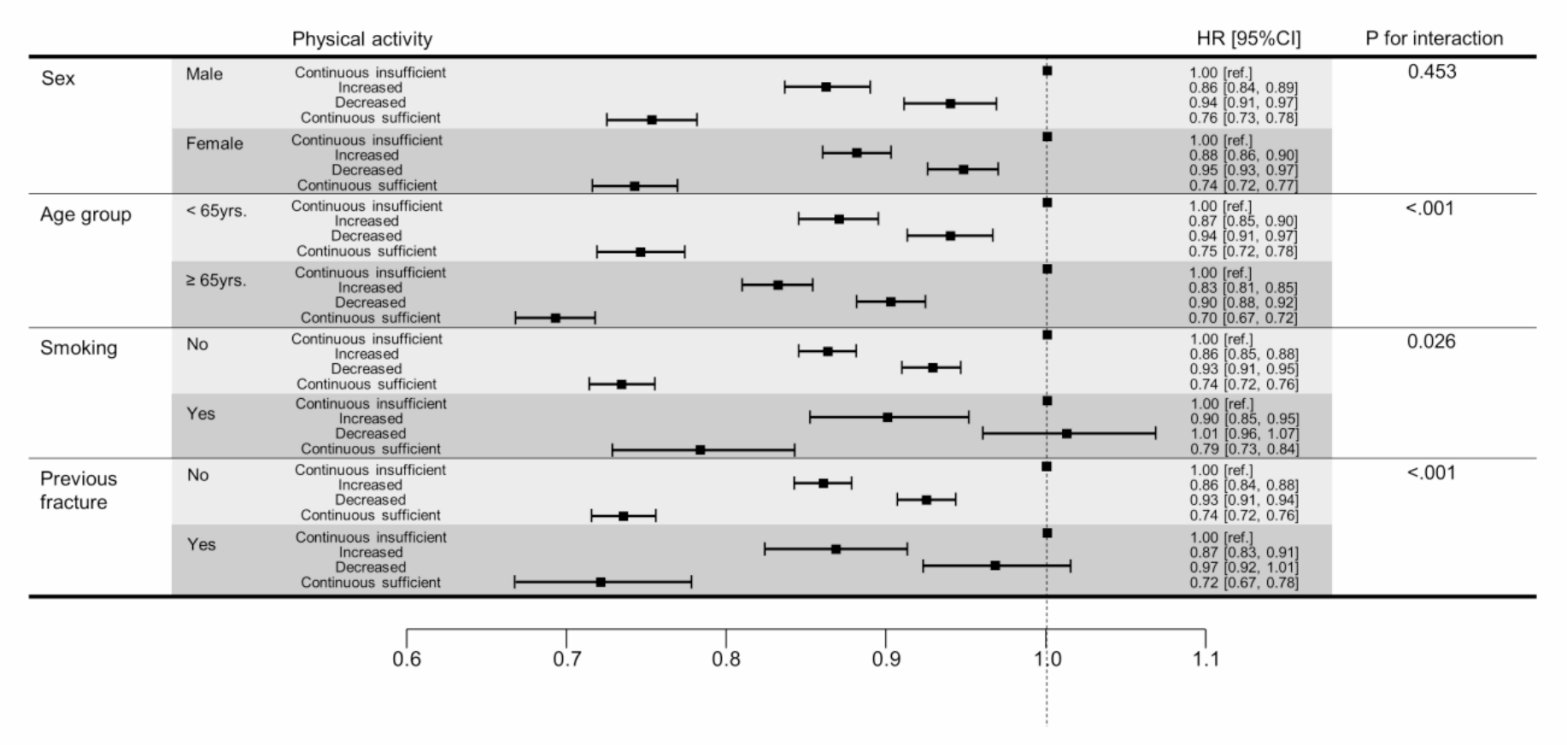
Subgroup analysis for association between RPA and vertebral fractures. HR, hazard ratio; CI, confidence interval

This study performed a subgroup analysis to determine the risk of vertebral fracture based on age, gender, smoking, drinking, household income, BMI, comorbidities (diabetes, dyslipidemia, CKD), and prior fracture. The p-value for interaction is an estimated value to evaluate the consistency of results within a subgroup. The exposure and subgroup multiplicative interaction term was added to the Cox proportional hazards regression model, and the p-value for that term was used. Participants with continuous sufficient physical activity who were aged 65 years or older, who did not smoke, and who were without prior fractures had a significantly reduced risk of vertebral fracture compared to participants with continuous insufficient physical activity. Sex, BMI, alcohol consumption, household income, and comorbidities appeared to have no significant effect (all *P* > 0.05). (Fig. 2) (sTable 1).

## Discussion

In this nationwide observational cohort study, we sought to determine the association between regular physical activity and vertebral fractures. The main finding was that participants who always had a sufficient level of physical activity had a lower risk of vertebral fracture than those who had insufficient physical activity, and the association was more significant in those over 65 years of age, non-smokers, and those without prior fractures.

Physical activity increases mechanical loading on bones, induces bone remodeling, strengthens bones, and develops muscles, improving quality of life by maintaining posture and improving function. Some studies have reported that physical activity can increase the risk of falling [26–28], it also helps to prevent falls and slips, preventing additional fractures or damage [13, 16]. Nonetheless, globally, one in four adults and three in four adolescents do not meet the level of physical activity recommended by the 2010 Global Recommendations on Physical Activity for Health, and these data have not improved over the years [29]. Physical activity can have various effects depending on intensity, type, and frequency. According to a previous study, no association was observed between leisure time activity level, intensity, or duration and the risk of vertebral fracture [14]. On the other hand, another study reported that moderate to vigorous activity reduces the incidence of vertebral fractures by about 33% [17]. These differences in results may be due to differences in regional and racial characteristics, as well as differences in how physical activity is defined or how vertebral fractures are defined. This study defined a vertebral fracture using the ICD-10 diagnosis code. Otherwise, previous study assessed vertebral fracture by dual-energy x-ray absorptiometry scans, and they reported physical activity does not tend to prevent vertebral fractures [14]. This study evaluated RPA using the IPAQ short version based on the dimensions (mode, frequency, duration, intensity), domains (occupational, domestic, transportation/utilitarian, leisure time), and decision matrix [18, 21]. A previous study validated the definition of RPA, but the study did not analyze vertebral fractures but hip fractures [30].

In addition, simple walking or low-impact activities do not have any particular effect on bone density, and resistance exercise is also reported to have no significant effect on bone density of the lumbar spine. Nonetheless, strength or resistance exercise may be helpful for people at risk for vertebral fractures because it can improve physical function [13]. This study showed that the Continuous Sufficient RPA group had a significantly reduced risk of vertebral fractures compared to the group without continuous sufficient RPA. It could be said that RPA plays a preventive role by compensating for factors such as falls, physical function decline, and neuromuscular problems that may be risk factors for fractures through exercise [13, 31].

Additionally, we confirmed that the preventive effect against vertebral fractures was more significant for people over 65 years of age. Recently, vertebral fractures have become recognized as a critical problem in the older population [32]. In older adults, vertebral fractures increase the risk of mortality as well as pain and physical impairment [33]. Therefore, it is important to prevent vertebral fractures by promoting muscle and physical ability through appropriate physical activity. However, high-intensity physical activity in older adults is likely to cause fragility fractures, so caution is required during physical activity, and it is essential to perform the exercise with an appropriate method and intensity tailored to each individual through specialists and supervision [13, 34, 35].

It was found that people who did not smoke had a lower risk of vertebral fractures. Smoking appears to have a direct toxic effect on bone osteoblasts and blood flow and causes problems in the physiological association of vitamin D and parathyroid hormone, making it a direct risk factor for fracture [36]. Nevertheless, a sufficient level of physical activity can be considered prophylactic because it has been shown to reduce the risk of vertebral fractures in our analysis, even in smokers.

Also, it was found that physical activity lowered the risk more significantly when there was no previous history of fractures. Once a fracture has occurred, the recovery process may be accompanied by pain and decreased physical activity, muscle mass, range of motion, and balance [37]. For these reasons, it is thought that qualitative differences in physical activity may occur in people with a history of fractures. Regardless, RPA lowers the risk of vertebral fractures in patients with and without a history of fractures. Therefore, to prevent vertebral fractures in clinical practice, it is necessary to establish a customized exercise plan according to each patient’s age, lifestyle, and history. There is clinical significance in that an appropriate level of RPA, not just pharmaceutical and invasive procedures, can play a significant role in preventing vertebral fractures, which are the leading causes of mobility and mortality. Therefore, physicians could anticipate positively affecting the patient’s outcome by identifying and providing education on an appropriate level of RPA when examining high-risk patients with vertebral fractures.

This study has the advantage of having an enormous sample size because it included data from nationwide examinations. This study is meaningful as it analyzed the association between RPA and vertebral fractures in a large number of participants. Additionally, previous studies have reported different contents depending on the definition of physical activity. This study evaluated physical activity using the IPAQ short version based on the dimensions (mode, frequency, duration, intensity), domains (occupational, domestic, transportation/utilitarian, leisure time), and decision matrix. Therefore, it could be said more systematic and consistent analysis of physical activity. However, this study had several limitations. First, because data from KNHIS were used, patients with vertebral fractures who did not use this medical system may have been inadvertently excluded. However, in Korea, 97% of the population is covered by this insurance system, so it can be considered a sufficiently representative analysis. Second, since the analysis was conducted on the Korean population, there may be limitations in generalizing it to other races, countries and regions. Third, data on physical activity were collected in a self-reported questionnaire, which could have introduced bias. However, we used the IPAQ score based on this, which has proven to be sufficiently valid in previous studies [38]. Fourth, the analysis was unavailable for fracture risk scores, history of drug use, bone density levels, or laboratory parameters that may be risk factors for fractures because the KNHIS database did not include such information. Also, mortality rates following fractures are not analyzed. Fifth, we only analyzed RPA. Given that the effects can vary depending on the intensity and frequency of exercise, caution is needed when applying the results of this study to actual clinical practice. Therefore, additional large-scale multi-ethnic and multi-national research will be needed to generalize the results of this study. Also, to clarify the mechanism between physical activity and vertebral fractures, future studies require additional analysis of bone density levels, fracture risk scores, laboratory parameters associated with fractures, and mortality rates following fractures.

## Conclusion

A sufficient level of RPA was found to be associated with a reduced risk of vertebral fractures and is more effective in people over 65 years of age, non-smokers, and those without prior fractures.

## Electronic Supplementary Material

Below is the link to the electronic supplementary material.

Supplementary Material 1

## Data Availability

Data available from the author (Jae-Young Hong; E-mail: osspine@korea.ac.kr) of this publication.
