# Supplementary material for: The impact of regular physical activity on vertebral fractures: Korean nationwide population-based cohort study
Source: BMC Musculoskelet Disord. 2025 Jan 22;26:78. doi: 10.1186/s12891-024-08179-2 (PMC11752946; doi:10.1186/s12891-024-08179-2)
Supplement: Supplementary file 1 — Supplementary Material 1 [file 12891_2024_8179_MOESM1_ESM.docx]

sTable 1. Subgroup analysis for vertebral fracture according to change in regular physical activity.

|  | **Regular PA** | **n** | **Fracture (n)** | **Hazard ratio**  **(95% CI)** | | **P for interaction** |
| --- | --- | --- | --- | --- | --- | --- |
| Sex | | | | | 0.453 | |
| Male | Continuous insufficient | 1687642 | 25587 | 1 | |  |
|  | Increased | 360244 | 4984 | 0.863 (0.838 - 0.89) | |  |
|  | Decreased | 322733 | 5247 | 0.94 (0.912 - 0.968) | |  |
|  | Continuous sufficient | 288410 | 3431 | 0.755 (0.728 - 0.782) | |  |
| Female | Continuous insufficient | 1615862 | 62674 | 1 | |  |
|  | Increased | 290644 | 8209 | 0.882 (0.862 - 0.902) | |  |
|  | Decreased | 260828 | 9055 | 0.948 (0.927 - 0.969) | |  |
|  | Continuous sufficient | 157781 | 3502 | 0.744 (0.719 - 0.77) | |  |
| Age | | | | | | <0.001 |
| < 65 yrs. | Continuous insufficient | 2680437 | 32735 | 1 | |  |
|  | Increased | 530886 | 5964 | 0.871 (0.847 - 0.895) | |  |
|  | Decreased | 458905 | 5832 | 0.94 (0.914 - 0.966) | |  |
|  | Continuous sufficient | 364257 | 3412 | 0.748 (0.722 - 0.775) | |  |
| ≥ 65 yrs. | Continuous insufficient | 623067 | 55526 | 1 | |  |
|  | Increased | 120002 | 7229 | 0.833 (0.812 - 0.854) | |  |
|  | Decreased | 124656 | 8470 | 0.903 (0.883 - 0.924) | |  |
|  | Continuous sufficient | 81934 | 3521 | 0.695 (0.671 - 0.719) | |  |
| Body Mass Index (kg/m^2^) | | | | | | 0.663 |
| < 25 | Continuous insufficient | 2182719 | 56911 | 1 | |  |
|  | Increased | 427395 | 8511 | 0.867 (0.848 - 0.887) | |  |
|  | Decreased | 371521 | 9052 | 0.932 (0.912 - 0.953) | |  |
|  | Continuous sufficient | 287145 | 4419 | 0.73 (0.708 - 0.753) | |  |
| ≥ 25 | Continuous insufficient | 1120785 | 31350 | 1 | |  |
|  | Increased | 223493 | 4682 | 0.867 (0.841 - 0.894) | |  |
|  | Decreased | 212040 | 5250 | 0.943 (0.916 - 0.971) | |  |
|  | Continuous sufficient | 159046 | 2514 | 0.761 (0.731 - 0.793) | |  |
| Smoking | | | | | | 0.026 |
| Non-Smoker | Continuous insufficient | 2602393 | 78335 | 1 | |  |
|  | Increased | 534829 | 11678 | 0.864 (0.847 - 0.881) | |  |
|  | Decreased | 478199 | 12664 | 0.929 (0.911 - 0.946) | |  |
|  | Continuous sufficient | 369779 | 6117 | 0.736 (0.717 - 0.756) | |  |
| Current Smoker | Continuous insufficient | 701111 | 9926 | 1 | |  |
|  | Increased | 116059 | 1515 | 0.901 (0.854 - 0.951) | |  |
|  | Decreased | 105362 | 1638 | 1.012 (0.961 - 1.067) | |  |
|  | Continuous sufficient | 76412 | 816 | 0.785 (0.731 - 0.843) | |  |
| Alcohol consumption | | | | | | 0.374 |
| No | Continuous insufficient | 1912011 | 69536 | 1 | |  |
|  | Increased | 356932 | 9465 | 0.863 (0.844 - 0.881) | |  |
|  | Decreased | 332570 | 10660 | 0.932 (0.913 - 0.951) | |  |
|  | Continuous sufficient | 214518 | 4459 | 0.736 (0.714 - 0.758) | |  |
| Yes | Continuous insufficient | 1391493 | 18725 | 1 | |  |
|  | Increased | 293956 | 3728 | 0.882 (0.851 - 0.914) | |  |
|  | Decreased | 250991 | 3642 | 0.956 (0.922 - 0.99) | |  |
|  | Continuous sufficient | 231673 | 2474 | 0.752 (0.721 - 0.785) | |  |
| Household income | | | | | | 0.281 |
| low 20% | Continuous insufficient | 2623691 | 68923 | 1 | |  |
|  | Increased | 513109 | 10285 | 0.864 (0.846 - 0.882) | |  |
|  | Decreased | 462227 | 11195 | 0.935 (0.917 - 0.954) | |  |
|  | Continuous sufficient | 364188 | 5505 | 0.732 (0.712 - 0.752) | |  |
| Other | Continuous insufficient | 679813 | 19338 | 1 | |  |
|  | Increased | 137779 | 2908 | 0.872 (0.838 - 0.906) | |  |
|  | Decreased | 121334 | 3107 | 0.937 (0.902 - 0.973) | |  |
|  | Continuous sufficient | 82003 | 1428 | 0.766 (0.726 - 0.809) | |  |
| Diabetes mellitus | | | | | | 0.131 |
| No | Continuous insufficient | 2925110 | 73917 | 1 | |  |
|  | Increased | 568594 | 10921 | 0.866 (0.849 - 0.884) | |  |
|  | Decreased | 503650 | 11737 | 0.936 (0.917 - 0.954) | |  |
|  | Continuous sufficient | 386675 | 5660 | 0.733 (0.713 - 0.753) | |  |
| Yes | Continuous insufficient | 378394 | 14344 | 1 | |  |
|  | Increased | 82294 | 2272 | 0.86 (0.823 - 0.899) | |  |
|  | Decreased | 79911 | 2565 | 0.93 (0.891 - 0.97) | |  |
|  | Continuous sufficient | 59516 | 1273 | 0.762 (0.72 - 0.808) | |  |
| History of fracture | | | | | | <0.001 |
| No | Continuous insufficient | 3203517 | 74390 | 1 | |  |
|  | Increased | 633356 | 11455 | 0.861 (0.844 - 0.878) | |  |
|  | Decreased | 566185 | 12221 | 0.925 (0.908 - 0.943) | |  |
|  | Continuous sufficient | 436043 | 6199 | 0.737 (0.718 - 0.757) | |  |
| Yes | Continuous insufficient | 99987 | 13871 | 1 | |  |
|  | Increased | 17532 | 1738 | 0.869 (0.826 - 0.913) | |  |
|  | Decreased | 17376 | 2081 | 0.968 (0.924 - 1.014) | |  |
|  | Continuous sufficient | 10148 | 734 | 0.723 (0.671 - 0.779) | |  |
| Chronic kidney disease | | | | | | 0.132 |
| No | Continuous insufficient | 3100191 | 77444 | 1 | |  |
|  | Increased | 610435 | 11800 | 0.864 (0.847 - 0.881) | |  |
|  | Decreased | 545391 | 12670 | 0.93 (0.913 - 0.948) | |  |
|  | Continuous sufficient | 418038 | 6223 | 0.732 (0.713 - 0.751) | |  |
| Yes | Continuous insufficient | 203313 | 10817 | 1 | |  |
|  | Increased | 40453 | 1393 | 0.867 (0.82 - 0.917) | |  |
|  | Decreased | 38170 | 1632 | 0.967 (0.917 - 1.019) | |  |
|  | Continuous sufficient | 28153 | 710 | 0.784 (0.726 - 0.847) | |  |

PA, physical activity; CI, Confidence interval
